# The Aseptic Closure of Long Standing Sinuses Having Their Origin in Tubercular Joints

**Published:** 1892-02

**Authors:** H. Augustus Wilson

**Affiliations:** Professor of General and Orthopedic Surgery in the Philadelphia Polyclinic and College for Graduates in Medicine; Cinical Professor of Orthopedic Surgery in the Woman’s Medical College of Pennsylvania; Clinical Lecturer on Orthopedic Surgery in the Jefferson Medical College of Philadelphia


### THE ASEPTIC CLOSURE OF LONG STANDING SINUSES HAVING THEIR ORIGIN IN TUBERCULAR JOINTS.*

BY H. AUGUSTUS WILSON, M. D.,
Professor of General and Orthopedic Surgery in the Philadelphia Polyclinic and College for Graduates in Medicine; Cinical Professor of Orthopedic Surgery in the Woman's Medical College of Pennsylvania; Clinical Lecturer on Orthopedic Surgery in the Jefferson Medical College of Philadelphia.

Recognizing the very extensive character of this subject, I have avoided elaborate details and arranged the paper more in the form of summary based upon modern aseptic practice, so that it should not occupy more time than is allowed for reading of papers. The class of cases that it is the purpose of this paper to discuss embraces a very large range of chronic runners from one hospital to another. They are usually designated as incurable or hopeless, and, as a consequence, are subjected to the so-called palliative or expectant plan of treatment, attention being largely confined to medication.

It occasionally happens that spontaneous resolution and closure of sinuses or fistulæ takes place, but this is the exception, the rule being that they continue patulous for a long time, often during the entire life of the patient. The well-recognized and thoroughly established fact that so-called cold abscesses frequently undergo absorption when unopened would seem to indicate the advisability of favoring such absorption by a closure of the openings that may have occurred from over accumulation.

The causes of these sinuses may be sought in a tubercular deposit in a bone or joint, or in the soft structures that surround a joint, which has formed a cold abscess, caseation and decomposition taking place, rupture follows. Part of the contents of the sac escapes and a sinus remains for a long period of years to act the part of a sewer-pipe.

* Read before the Philadelphia Academy of Surgery November 2, 1891. Reprint.

When rupture does not take place spontaneously it is apt to be induced by the evacuation of a cold abscess by an aspirator, for in this procedure it rarely occurs that the entire deposit is removed because it is not of the nature of a fluid. In the closure of the skin-wound made by the aspirator, the cicatrix is only superficial, and the subsequent spontaneous rupture is therefore facilitated at the site of puncture.

Likewise after incision, when the contents of the sac are thoroughly removed, the too long continued use of the drainage tube, or in some instances its use at all produces a sinus by the separation of tissues that would otherwise granulate. This sinus has not only no tendency to close, but the oft-repeated injections for the purpose of rendering the parts aseptic interferes with any granulation process that may have been commenced. In these ways sinuses are formed which persist, although often subjected to prolonged medication because of the supposed danger to the patient of any radical attempt at closure.

The teaching of Gross is still observed. "When the fistula has been of long standing, and has acted all along as a drain upon the system, serving perhaps to counteract some other affection, such as phthisis, or a tendency to apoplexy, no operation should be practiced, since it could hardly fail to provoke mischief ; in fact, serious organic disease of any kind is a contraindication to an operation. The only exception to this is where the fistula is a cause of excessive local distress, completely depriving the patient of sleep, appetite and comfort. Under such circumstances the surgeon could hardly refuse his aid, but before doing this, he would be sure to open a new course of counter-irritation, in the form of an issue or seton, in some other or more eligible portion of the body, thus establishing a drain at least equal to that which he is about to suppress as a means of temporary mitigation."

In marked contrast is the modern teaching, for since the above was written in 1872, the adoption of aseptic methods has made it possible to reverse entirely the plan of procedure.

It is often best to consider a tubercular focus to be a malignant growth tending to increased destruction if undisturbed. While it is not malignant *per se* in the sense of malignant tumors tending to the death of the patient, radical measures will more frequently and successfully be resorted to if this view is kept in mind in preference to its harmless character.

The freedom from disastrous results, in fact, the satisfactory recoveries obtained in excisions of tubercular hip disease, knee-joint involvements, and even when vertebræ are attacked, all tend to urge the adoption of this plan of procedure in the early stages of the disease, to limit the extent of the excision to the minimum.

Excision is not confined to early stages, but is as well adapted to conditions where the necrosis is very extensive and is a procedure now well established, but it not infrequently happens that a sinus follows which could have been avoided by recourse to methods to be alluded to later.

The vicious character of the infected parts tends to their non-union, and every means that can safely be resorted to for the complete closure of long-standing sinuses should be resorted to.

The great difficulty that is experienced of tracing a sinus after the parts have been laid open may be met in two ways. Prior to opening the sinus a probe may be introduced to the furthermost part and allowed to remain as a guide.

The injection into the sinus and cavities in connection therewith of some coloring matter which will be innocuous, and at the same time so stain the lining membrane that its discernment and quick removal may be facilitated. I have found that a solution of pyoktanin meets the indications efficiently, for it possesses germicidal properties, and the greatest objection to its more general use is here its highest recommendation, for its purple color stains the tissues with which it comes in contact, thereby clearly indicating the tissues that it is desirable to remove. The object to be sought is the entire removal by clean incision of all of the stained tissue or lining membrane of the sinuses, and when the site of the original tubercular deposit is reached to excise it completely.

The laceration of tissues by tearing as a result of the use of an ecraseur or dry dissector or handle of a knife tends to sloughing, and the necessity of providing an outlet by drainage tube, the avoidance of which is of considerable importance.

The infection of freshly incised tissue by the bacillus tuberculosa may be avoided by the free use of irrigation of sterilized water or solution 1 to 2,000 bichloride of mercury during the progress of the operation and the efficient use of iodoform before closure.

It not infrequently happens that a suspected bone origin to a sinus is found upon laying open the parts not to exist, but that the tubercular deposit is confined to soft structures and its ready removal easily accomplished.

In cases where a bone is found to be involved, the removal of the necrosed or diseased part should be done by a chisel, to the end that only normal tissue be allowed to remain. The process of superficially scraping is inadequate for the entire removal of the diseased tissues, and by its laceration does not conduce to healthy cicatrization.

If one or more contiguous bones are partially involved, the entire removal of such bones or of the joints is not essential, but only of such parts as are involved. To let the sinuses alone, or to continue the expectant plan of treatment, means a continuance of the annoyance of dribbling pus, positive discomfort, and a constant menace to the general health with but slight tendency towards recovery.

The constitutional disturbance depends more largely upon the exudation from the lining membrane of the sinus than from the tubercular deposit, as evidenced by the frequent freedom from constitutional disturbance in cases of unopened cold abscesses, and by the very great improvement in the general health following the successful closure of those sinuses which have existed for a long period.

The simple injection plan of treatment is found usually to be inefficient because of the mechanical difficulty of covering the entire surface of a sac by any material thrown in through a single opening, without recourse to hyperdistension. The danger of internal rupture of the sac at some weak and inaccessible point by hyperdistension is very great, and when this does take place not only the material injected is thrown outside of the sac, but a new field of absorbent vessels is exposed to infection of bacilli.

A vent hole or counter-opening at the opposite side or furthermost end of the sac or sinus avoids the danger of rupture and acts like a check valve, enabling the operator to command the quantity of material injected as well as the force of the flow.

Sinuses of great length may be closed by stages where the discharge is considerable by substituting an opening nearer the focus, thereby diminishing the constitutional effect of exudation from the greater surface and the portion between the openings completely closed. In turn this may often be still further shortened, until finally a complete closure is accomplished.

The danger of stitch wound abscess and the unsightly transverse cicatrices, which is very considerable in these cases, may best be avoided by having the sutures embrace only subcutaneous tissues, bringing the needle out through the edge of the incision and not through the skin. By this means deeper union is induced and the possibility of any gaping of the skin is avoided by the use of collodion-saturated gauze covering the incision.

Iodoform is pre-eminently a germicide for bacillus tuberculosus, and it is of great value in making it possible to seal the wound. The form most satisfactory for use in these cases being a 10 per cent. emulsion in freshly-boiled olive oil. The dry powder may be used, but its even distribution is difficult to accomplish and the crevices are not reached. The etherial solution has been found objectionable, on the account of its too rapid absorption and the danger of iodoform intoxication.

The resort to packing with lint or other substance for the purpose of keeping the skin wound open and to induce granulation from the bottom, as well as the use of any kind of drainage, is generally unecessary and often positively harmful, in favoring a continuance of the sinus.

If the parts are cleanly incised and maintained in close approximation, primary union may be expected throughout, and, if only healthy tissue be allowed to remain, drainage need not be employed. Occasionally it may be deemed expedient to use drainage for the first twenty-four hours, in cases where the pus continues to flow from inaccessible points, but its continued use is disadvantageous.

The procedures to be adopted may best be considered if the conditions are grouped as follows:

1. Those sinuses in connection with accessible joints where the tubercular deposit can be safely removed.

2. In similar positions, but where its removal cannot be safely accomplished.

3. Sinuses from inaccessible deposits.

Under the first heading, sinuses in connection with accessible joints, where the tubercular deposit can be safely removed, the modern plan of procedure is self-evident. Under strict asepsis, or chemical anti-sepsis, the focus should be removed in its entirety, leaving only healthy tissue behind. The cavity of the sinus denuded of its lining membrane by clean incision, in preference to tearing or scraping, and the entire cavity of the sinus and of the site of the former deposit rendered aseptic by thorough washing with peroxide of hydrogen, followed by irrigation of 1 to 2,000 bichloride of mercury, and, finally, the entire surface covered with iodoform emulsion. The parts are then to be brought into coaptation by subcutaneous sutures; iodoform dusted over incision; collodion gauze; finally, hermetically sealing the wound. Gentle but firm pressure with aseptic gauze and bandages complete the dressings.

II. Where the sinuses are in connection with accessible joints, where the removal of the tubercular deposit cannot be safely accomplished.

In these cases, as, for example, in hip disease, when the ilium has become denuded or involved, or in the lumbar vertebræ, it has been found judicious surgery to cut away all that could safely be removed, washing the parts as thoroughly as though the entire removal had been accomplished, as referred to under the first heading, and sealing the wound as described.

It will be expected that new cold abscesses will form from the unremoved unhealthy tissue, necessitating reopening, and the probability of this should be placed before the patient, so that at the very first indication of the necessity, the former procedure should be repeated. The relief afforded by a cessation of the annoyances of the sinuses will more than compensate for the possibility of repeating the operation, nor is it certain that repetition will really be necessary.

III. Where the sinuses have their origin in inaccessible deposits—for example, when the bodies of the dorsal vertebræ are involved—it is often clearly impossible to lay open the sinus or reach the site of deposit, and recourse must, therefore, be had to other but less satisfactory means.

In most of these cases, the sinus only can be considered, and remedial measures must be confined to injections to render the parts thoroughly aseptic. A counter-opening, when practicable, greatly facilitates the accomplishment of the desired end—in fact, is often really indispensable. The closure of the sinus may be facilitated by excising as much of the outlet as possible, so as to procure union to a greater depth than by simply closing the skin opening. Both openings being closed, pressure is to be relied upon to close the sac. It is possible that in the attempt to eradicate the bacilli and effects from the sinus that the injected germicide may reach the site of the deposit, and act directly upon the focus, in which case the permanent benefit will be great.

In the cases upon which I have thus operated I have had no re-opening, or constitutional or other disturbances follow, but the time that has elapsed since the operations were performed is entirely too short to afford any indication of the permanence of the results obtained.

To have closed and kept closed for a year a sinus of the hip-joint of twenty-three years' standing is enough encouragement for a continuance of the method. To have removed a drainage tube from a knee that had been in constant use for eighteen months, the sinus having been daily subjected to washing, and new external dressings employed to catch the pus that should not have been allowed to continue to flow, and to have closed the sinus and have it remain healthy for nearly six months, is also encouraging.

It is my purpose to detail the results in these cases when sufficient time has elapsed to warrant the statement that they are permanently benefited. The full purpose of this paper will have been met if it assists in any way in the judicious treatment o a most troublesome class of cases.

1611 *Spruce Street.*

